# T2 mapping vs T2 weighted imaging in the detection of myocardial oedema

**DOI:** 10.1186/1532-429X-14-S1-P266

**Published:** 2012-02-01

**Authors:** Elisa McAlindon, Peter Weale, Jessica Harris, David Smith, Andreas Baumbach, Tom Johnson, Chiara Bucciarelli-Ducci

**Affiliations:** 1 Bristol Heart Institute, NIHR Cardiovascular BRU, Bristol, UK; 2 Siemens Medical Solutions, London, UK

## Background

The “gold standard” CMR sequence for assessing the myocardial oedema or area at risk following an acute coronary syndrome is controversial. T2 Short Tau Inversion Recovery (T2-STIR) is in widespread clinical use but can lack robustness. Steady state free precession oedema imaging (SSFP/ ACUT2E) has emerging data to support it as a more reproducible method for area at risk (AAR) assessment. We tested a novel T2 mapping method to AAR. The potential benefit of this method is that the numerical output of the method is largely independent of myocardial motion, instrumental errors (eg surface coil normalisation methods). The aim of this study was to compare the novel T2 mapping method with the two existing methods of assessing AAR (T2-STIR and SSFP/ACUT2E).

## Methods

30 slices in 10 patients day 2-4 following acute myocardial infarction were analysed by 3 sequences (T2-STIR, ACUT2E, and T2 mapping). The images were analysed using a semi-automated software, and the AAR was expressed as a % of total slice area. The window setting was defined as the sum of the mean signal intensity (SI) of the unaffected area plus 2 standard deviation (SD) for this area. The level setting was set at the mean SI of the unaffected area (a method used in previous published studies). Inter-method and inter-observer variability was assessed using the Bland Altman method. Qualitative inter-observer and inter-method variability was assessed: each slice split into segments according to the 17 segment model and oedema in each segment scored as present of absent.

## Results

On the Bland Altman plots we observed a better agreement between for T2-STIR vs T2 map, than ACUT2E vs T2 map (Image 1).

**Figure 1:**
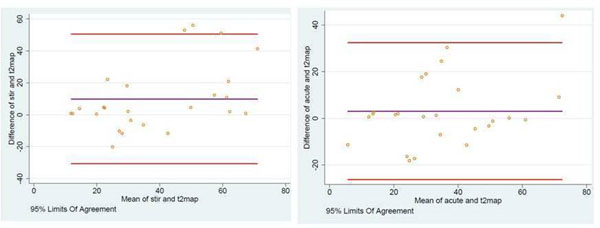
Bland Altman plots for AAR assessed by T2 mapping (t2map) vs STIR (left panel) and SSFP (acute)(right B).

**Figure 2:**
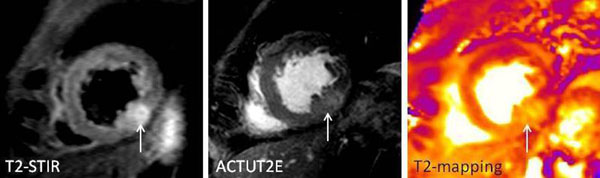
Short-axis slice repeated with the 3 techniques showing an oedematous area in the apical inferior wall.

On qualitative assessment, there is very good agreement between T2-STIR and T2 map (kappa 0.71, 86% segments agree) and ACUT2E and T2 map (kappa 0.81, 91% segments agree). We also found a good agreement between T2-STIR and ACUT2E (kappa 0.78, 89% segments agree).

On assessing qualitative inter-observer reproducibility, there is a good agreement between observers using all 3 sequences; T2-STIR appears to have the lowest interobserver agreement (T2-STIR kappa 0.56, ACUT2E kappa 0.67, T2 map kappa 0.67).

## Conclusions

T2 mapping method may provide a viable alternative to current AAR methods. This needs to be further assessed in a larger patient population.

## Funding

NIHR Cardiovascular BRU, Bristol Heart Institute.

